# Bis[μ-1-(2-pyridylmeth­yl)-1*H*-benzo­triazole]disilver(I) bis­(perchlorate)

**DOI:** 10.1107/S1600536809043669

**Published:** 2009-10-28

**Authors:** Chun-Sen Liu, Xian-Guo Xiao, Min Hu

**Affiliations:** a Zhengzhou University of Light Industry, Henan Provincial Key Laboratory of Surface & Interface Science, Henan, Zhengzhou 450002, People’s Republic of China; b College of Mechanical Engineering, Zhengzhou University, Henan, Zhengzhou 450001, People’s Republic of China

## Abstract

In the title centrosymmetric binuclear Ag^I ^complex, [Ag_2_(C_12_H_10_N_4_)_2_](ClO_4_)_2_, each Ag^I^ center is two-coordinated by one pyridine and one benzotriazole *N*-donor atom of two inversion-related 1-(2-pyridylmeth­yl)-1*H*-benzotriazole (*L*) ligands. This forms a unique box-like cyclic dimer with an intra­molecular Ag⋯Ag separation of 4.479 (2) Å. Inter­molecular C—H⋯O hydrogen-bonding inter­actions, involving uncoordinated ClO_4_^−^ ions, link the binuclear units, forming a two-dimensional network parallel to (10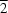).

## Related literature

Bis-heterocyclic chelating or bridging ligands have been used extensively to construct functional coordination complexes that contain different hetero-aromatic ring systems, see: Constable (1989 ▶); Constable & Steel (1989 ▶); Steel (2005 ▶). For related structures, see: Hu *et al.* (2008 ▶); Huang *et al.* (2008 ▶); Liu *et al.* (2006 ▶, 2007 ▶); Liu, Sun *et al.* (2008 ▶); Liu, Zhou *et al.* (2008 ▶); Richardson & Steel (2003 ▶). For the synthesis of ligand *L*, see: Liu, Sun *et al.* (2008 ▶). 
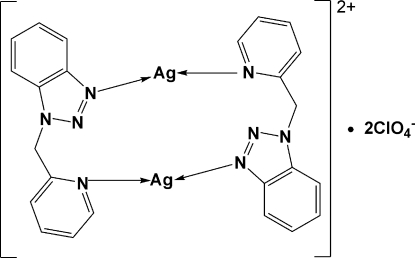


## Experimental

### 

#### Crystal data

[Ag_2_(C_12_H_10_N_4_)_2_](ClO_4_)_2_
*M* *_r_* = 835.12
Monoclinic, 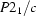

*a* = 9.4273 (4) Å
*b* = 16.0863 (7) Å
*c* = 11.9152 (7) Å
β = 128.448 (3)°
*V* = 1415.15 (13) Å^3^
*Z* = 2
Mo *K*α radiation
μ = 1.64 mm^−1^
*T* = 293 K
0.24 × 0.23 × 0.03 mm

#### Data collection

Bruker SMART CCD area-detector diffractometer
Absorption correction: multi-scan (*SADABS*; Sheldrick, 1996 ▶) *T*_min_ = 0.695, *T*_max_ = 0.960
9388 measured reflections
2484 independent reflections
1658 reflections with *I* > 2σ(*I*)
*R*_int_ = 0.037

#### Refinement

*R*[*F* ^2^ > 2σ(*F* ^2^)] = 0.043
*wR*(*F* ^2^) = 0.137
*S* = 1.08
2484 reflections
199 parameters
H-atom parameters constrained
Δρ_max_ = 0.62 e Å^−3^
Δρ_min_ = −0.53 e Å^−3^

### 

Data collection: *SMART* (Bruker, 2007 ▶); cell refinement: *SAINT* (Bruker, 2007 ▶); data reduction: *SAINT*; program(s) used to solve structure: *SHELXS97* (Sheldrick, 2008 ▶); program(s) used to refine structure: *SHELXL97* (Sheldrick, 2008 ▶); molecular graphics: *SHELXTL* (Sheldrick, 2008 ▶); software used to prepare material for publication: *SHELXTL* and *PLATON* (Spek, 2009 ▶).

## Supplementary Material

Crystal structure: contains datablocks I, global. DOI: 10.1107/S1600536809043669/zq2014sup1.cif

Structure factors: contains datablocks I. DOI: 10.1107/S1600536809043669/zq2014Isup2.hkl

Additional supplementary materials:  crystallographic information; 3D view; checkCIF report

## Figures and Tables

**Table:** 

Ag1—N2^i^	2.159 (5)
Ag1—N1	2.201 (5)

**Table:** 

N2^i^—Ag1—N1	155.9 (2)

Symmetry code: (i) 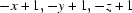.

**Table 2:** Hydrogen-bond geometry (Å, °)

*D*—H⋯*A*	*D*—H	H⋯*A*	*D*⋯*A*	*D*—H⋯*A*
C1—H11⋯O3^i^	0.97	2.31	3.264 (15)	168
C1—H12⋯O2^ii^	0.97	2.58	3.415 (11)	144
C3—H3⋯O1^ii^	0.93	2.60	3.512 (10)	168
C11—H1⋯O1^ii^	0.93	2.56	3.481 (11)	170

Symmetry codes: (i) 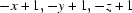; (ii) 
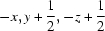
.

**Fig. 1.**
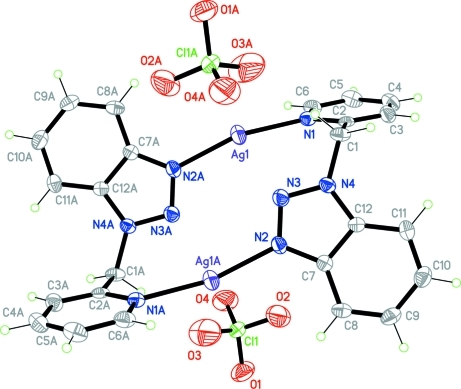
The molecular structure of complex (I). Displacement ellipsoids are drawn at the 30% probability level. Atoms labelled with the suffix A are generated by the symmetry operation (–x + 1, –y + 1, –z + 1).

**Fig. 2.**
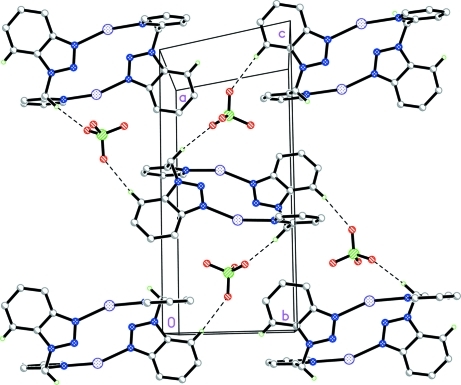
A view of the two-dimensional network of complex (I), running parallel to the (102) plane, formed by the intermolecular C—H···O (fine dashed lines) interactions. For clarity, only H atoms involved in the interactions are shown.

## supplementary crystallographic information

### Comment

Numerous related bis-heterocyclic chelating or bridging ligands have been synthesized and used extensively to construct functional coordination complexes that contain different hetero-aromatic ring systems, for example, pyridine, pyrazine, quinoline, quinoxaline, pyrazole, imidazole, thiazoles and their benzo analogues (Constable, 1989; Constable & Steel, 1989; Steel, 2005). The structures of five N-containing bis-heterocyclic ligands bearing 1-substituted benzotriazole subunits, such as 1-(2-pyridylmethyl)-1*H*-benzotriazole and its Ru^II^, Cu^II^, Pd^II^, Ag^I^, Zn^II^ and Hg^II^ complexes, have been well documented previously (Huang *et al.* 2008; Liu, Zhou *et al.* 2008; Richardson & Steel, 2003). In our previous work, to further investigate the influences of the N-donor spatial position of pendant pyridyl group in structurally related benzotriazol-1-yl-based pyridyl ligands on the structures of their coordination complexes, two new N-containing heterocyclic ligands 1-(4-pyridylmethyl)-1*H*-benzotriazole (4-pbt) and 2-(3-pyridylmethyl)-2*H*-benzotriazole (3-pbt) were designed and prepared. Their reaction with AgNO_3_ offered an one-dimensional double helical coordination polymer {[Ag(4-pbt)](NO_3_)}_∞_ and a centrosymmetric binuclear complex [Ag_2_(3-pbt)_2_ (NO_3_)_2_], respectively (Hu *et al.* 2008; Liu, Sun *et al.* 2008). To further investigate the influence of different counter-anions on the self-assembly process of coordination complexes, we chose to use *L* to construct new functional Ag^I^ complexes through its reaction with AgClO_4_. Here we report the crystal structure of [Ag(*L*)]_2_(ClO_4_)_2_.

The structure of the title compound (I) consists of a centrosymmetric binuclear [Ag(*L*)]_2_^2+ ^unit and two uncoordinated ClO_4_^-^ ions. The binuclear [Ag(*L*)]_2_^2+ ^cation (Fig. 1) comprises two *L *ligands and two Ag^I^ centers. The intramolecular non-bonding Ag···Ag separation is 4.479 (2) Å. There is only one crystallographic independent Ag^I ^center, which is two coordinated by two N-atom donors, one N donor being from benzotriazole ring of one *L* ligand and the other being from pyridyl ring of another *L* ligand. In this case the 14-membered dimetallocyclic ring is far from planar as a result of the presence of the tetrahedral methylene group of the *L *ligand. All the Ag—N bond distances are in the normal range found for similar complexes (Liu *et al.*, 2006; Liu *et al.*, 2007).

In the crystal structure adjacent discrete binuclear [Ag(*L*)]_2_^2+^ units are assembled into one-dimensional chains by intermolecular C—H···O hydrogen-bonding interactions between the *L *ligands and the uncoordinated ClO_4_^- ^(Table 2). The net result is a two-dimensional network running parallel to the (102) plane (Fig. 2). In addition, the crystal structure of (I) also contains intermolecular face-to-face π···π stacking interactions between the pyridyl ring involving N1/C2/C3/C4/C5/C6 (centroid *Cg*1) and N1A/C2A/C3A/C4A/C5A/C6A (centroid *Cg*2) of distinct *L* ligands [the centroid–centroid separation being 3.685 (1) Å, symmetry code A: -*x*, -*y* + 1, -*z *+ 1], that interlink the two-dimensional sheets to form a three-dimensional framework.

### Experimental

1-(2-Pyridylmethyl)-1*H*-benzotriazole (*L*) was synthesized according to the literature procedure of Liu, Sun *et al.* (2008). Complex (I) was prepared by adding a solution of *L* (0.05 mmol) in CH_3_OH (10 ml) on top of an aqueous solution (15 ml) of AgClO_4_ (0.1 mmol) in a test tube. Yellow single crystals suitable for X-ray structural analysis appeared at the tube wall after *ca* one month at room temperature (yield ~30% based on *L*). Elemental analysis calculated for (C_24_H_20_Ag_2_Cl_2_N_8_O_8_): H 2.41, C 34.52, N 13.42%; found: H 2.30, C 34.67, N 13.36%.

### Refinement

H atoms were included in calculated positions and treated in the subsequent refinement as riding atoms, with C—H = 0.93 (aromatic) or 0.97 Å (methylene), with *U*_iso_(H) = 1.2 *U*_eq_(C).

### Crystal data

**Table:**

[Ag_2_(C_12_H_10_N_4_)_2_](ClO_4_)_2_	*F*(000) = 824
*M**_r_* = 835.12	*D*_x_ = 1.960 Mg m^−^^3^
Monoclinic, *P*2_1_/*c*	Mo *K*α radiation, λ = 0.71073 Å
Hall symbol: -P 2ybc	Cell parameters from 3182 reflections
*a* = 9.4273 (4) Å	θ = 3.0–26.3°
*b* = 16.0863 (7) Å	µ = 1.64 mm^−^^1^
*c* = 11.9152 (7) Å	*T* = 293 K
β = 128.448 (3)°	Block, yellow
*V* = 1415.15 (13) Å^3^	0.24 × 0.23 × 0.03 mm
*Z* = 2	

### Data collection

**Table:**

Bruker SMART CCD area-detector diffractometer	2484 independent reflections
Radiation source: fine-focus sealed tube	1658 reflections with *I* > 2σ(*I*)
graphite	*R*_int_ = 0.037
φ and ω scans	θ_max_ = 25.0°, θ_min_ = 3.0°
Absorption correction: multi-scan (*SADABS*; Sheldrick, 1996)	*h* = −11→11
*T*_min_ = 0.695, *T*_max_ = 0.960	*k* = −15→19
9388 measured reflections	*l* = −13→14

### Refinement

**Table:**

Refinement on *F*^2^	Primary atom site location: structure-invariant direct methods
Least-squares matrix: full	Secondary atom site location: difference Fourier map
*R*[*F*^2^ > 2σ(*F*^2^)] = 0.043	Hydrogen site location: inferred from neighbouring sites
*wR*(*F*^2^) = 0.137	H-atom parameters constrained
*S* = 1.08	*w* = 1/[σ^2^(*F*_o_^2^) + (0.0614*P*)^2^ + 2.0392*P*] where *P* = (*F*_o_^2^ + 2*F*_c_^2^)/3
2484 reflections	(Δ/σ)_max_ = 0.001
199 parameters	Δρ_max_ = 0.62 e Å^−^^3^
0 restraints	Δρ_min_ = −0.53 e Å^−^^3^

### Special details

**Table:**

Geometry. All e.s.d.'s (except the e.s.d. in the dihedral angle between two l.s. planes) are estimated using the full covariance matrix. The cell e.s.d.'s are taken into account individually in the estimation of e.s.d.'s in distances, angles and torsion angles; correlations between e.s.d.'s in cell parameters are only used when they are defined by crystal symmetry. An approximate (isotropic) treatment of cell e.s.d.'s is used for estimating e.s.d.'s involving l.s. planes.
Refinement. Refinement of *F*^2^ against ALL reflections. The weighted *R*-factor *wR* and goodness of fit *S* are based on *F*^2^, conventional *R*-factors *R* are based on *F*, with *F* set to zero for negative *F*^2^. The threshold expression of *F*^2^ > σ(*F*^2^) is used only for calculating *R*-factors(gt) *etc*. and is not relevant to the choice of reflections for refinement. *R*-factors based on *F*^2^ are statistically about twice as large as those based on *F*, and *R*- factors based on ALL data will be even larger.

### Fractional atomic coordinates and isotropic or equivalent isotropic displacement parameters (Å^2^)

**Table:**

	*x*	*y*	*z*	*U*_iso_*/*U*_eq_	
Ag1	0.42617 (7)	0.60201 (4)	0.58311 (7)	0.0599 (3)	
N1	0.1418 (7)	0.6101 (3)	0.4978 (6)	0.0368 (13)	
N2	0.3065 (6)	0.4519 (4)	0.2962 (5)	0.0385 (14)	
N3	0.2868 (7)	0.5274 (4)	0.3285 (6)	0.0407 (14)	
N4	0.1091 (6)	0.5452 (3)	0.2391 (5)	0.0347 (13)	
C1	0.0453 (9)	0.6239 (4)	0.2566 (7)	0.0403 (17)	
H11	0.1386	0.6658	0.2933	0.048*	
H12	−0.0612	0.6425	0.1637	0.048*	
C2	−0.0004 (9)	0.6159 (4)	0.3575 (7)	0.0325 (15)	
C3	−0.1725 (9)	0.6158 (4)	0.3083 (8)	0.0435 (18)	
H3	−0.2665	0.6190	0.2102	0.052*	
C4	−0.2110 (10)	0.6110 (4)	0.4035 (9)	0.051 (2)	
H4	−0.3297	0.6105	0.3711	0.062*	
C5	−0.0673 (11)	0.6068 (4)	0.5464 (9)	0.052 (2)	
H5	−0.0869	0.6050	0.6138	0.063*	
C6	0.1070 (10)	0.6055 (4)	0.5901 (8)	0.0472 (18)	
H6	0.2035	0.6012	0.6874	0.057*	
C7	0.1415 (8)	0.4210 (4)	0.1852 (6)	0.0318 (15)	
C8	0.0907 (9)	0.3446 (4)	0.1149 (7)	0.0400 (17)	
H8	0.1757	0.3039	0.1390	0.048*	
C9	−0.0903 (10)	0.3326 (5)	0.0089 (8)	0.0500 (19)	
H9	−0.1290	0.2828	−0.0419	0.060*	
C10	−0.2202 (10)	0.3928 (5)	−0.0261 (8)	0.052 (2)	
H10	−0.3421	0.3811	−0.0980	0.062*	
C11	−0.1737 (8)	0.4676 (5)	0.0417 (7)	0.0407 (17)	
H1	−0.2595	0.5076	0.0183	0.049*	
C12	0.0111 (8)	0.4803 (4)	0.1486 (6)	0.0310 (14)	
Cl1	0.4844 (2)	0.21950 (12)	0.5792 (2)	0.0510 (5)	
O1	0.4745 (9)	0.1317 (4)	0.5670 (7)	0.089 (2)	
O2	0.3143 (9)	0.2532 (5)	0.4653 (7)	0.116 (3)	
O3	0.6166 (13)	0.2454 (6)	0.5735 (14)	0.168 (5)	
O4	0.5263 (10)	0.2432 (5)	0.7115 (7)	0.109 (3)	

### Atomic displacement parameters (Å^2^)

**Table:**

	*U*^11^	*U*^22^	*U*^33^	*U*^12^	*U*^13^	*U*^23^
Ag1	0.0348 (3)	0.0672 (5)	0.0583 (4)	0.0086 (3)	0.0195 (3)	−0.0078 (3)
N1	0.041 (3)	0.034 (3)	0.038 (3)	0.002 (2)	0.026 (3)	−0.003 (3)
N2	0.033 (3)	0.047 (4)	0.032 (3)	0.007 (3)	0.019 (2)	0.002 (3)
N3	0.037 (3)	0.045 (4)	0.035 (3)	0.004 (3)	0.019 (3)	−0.002 (3)
N4	0.037 (3)	0.036 (3)	0.034 (3)	0.006 (2)	0.023 (2)	0.000 (3)
C1	0.059 (4)	0.026 (4)	0.040 (4)	0.006 (3)	0.033 (4)	0.004 (3)
C2	0.044 (4)	0.017 (3)	0.032 (3)	0.003 (3)	0.021 (3)	−0.003 (3)
C3	0.039 (4)	0.046 (5)	0.038 (4)	0.008 (3)	0.020 (3)	0.004 (3)
C4	0.050 (4)	0.049 (5)	0.064 (5)	0.010 (3)	0.040 (4)	0.008 (4)
C5	0.074 (6)	0.046 (5)	0.061 (5)	0.012 (4)	0.054 (5)	0.012 (4)
C6	0.057 (4)	0.036 (4)	0.043 (4)	0.008 (3)	0.028 (4)	0.001 (3)
C7	0.036 (3)	0.034 (4)	0.026 (3)	0.004 (3)	0.020 (3)	0.006 (3)
C8	0.055 (4)	0.032 (4)	0.043 (4)	0.005 (3)	0.036 (4)	0.001 (3)
C9	0.058 (5)	0.036 (4)	0.052 (5)	−0.011 (4)	0.032 (4)	−0.009 (4)
C10	0.043 (4)	0.051 (5)	0.050 (4)	−0.011 (4)	0.023 (4)	−0.001 (4)
C11	0.033 (3)	0.050 (5)	0.037 (4)	0.005 (3)	0.021 (3)	0.008 (3)
C12	0.039 (3)	0.028 (4)	0.032 (4)	0.000 (3)	0.025 (3)	0.002 (3)
Cl1	0.0455 (9)	0.0446 (11)	0.0560 (12)	−0.0073 (8)	0.0281 (9)	−0.0116 (9)
O1	0.094 (4)	0.051 (4)	0.090 (5)	−0.012 (3)	0.041 (4)	−0.011 (3)
O2	0.098 (5)	0.117 (6)	0.066 (5)	0.042 (5)	0.018 (4)	−0.015 (4)
O3	0.171 (8)	0.115 (7)	0.317 (14)	−0.064 (6)	0.201 (10)	−0.063 (8)
O4	0.114 (5)	0.116 (6)	0.060 (4)	0.019 (5)	0.037 (4)	−0.028 (4)

### Geometric parameters (Å, °)

**Table:**

Ag1—N2^i^	2.159 (5)	C5—C6	1.381 (11)
Ag1—N1	2.201 (5)	C5—H5	0.9300
N1—C6	1.332 (9)	C6—H6	0.9300
N1—C2	1.347 (8)	C7—C12	1.394 (8)
N2—N3	1.322 (8)	C7—C8	1.394 (9)
N2—C7	1.362 (8)	C8—C9	1.365 (9)
N2—Ag1^i^	2.159 (5)	C8—H8	0.9301
N3—N4	1.344 (7)	C9—C10	1.406 (10)
N4—C12	1.367 (8)	C9—H9	0.9300
N4—C1	1.470 (8)	C10—C11	1.361 (10)
C1—C2	1.514 (10)	C10—H10	0.9300
C1—H11	0.9698	C11—C12	1.393 (8)
C1—H12	0.9699	C11—H1	0.9300
C2—C3	1.339 (9)	Cl1—O3	1.355 (7)
C3—C4	1.392 (11)	Cl1—O2	1.414 (6)
C3—H3	0.9300	Cl1—O1	1.417 (6)
C4—C5	1.367 (11)	Cl1—O4	1.418 (7)
C4—H4	0.9299		
			
N2^i^—Ag1—N1	155.9 (2)	C6—C5—H5	120.2
C6—N1—C2	117.5 (6)	N1—C6—C5	122.3 (7)
C6—N1—Ag1	118.1 (5)	N1—C6—H6	118.8
C2—N1—Ag1	124.3 (5)	C5—C6—H6	118.9
N3—N2—C7	109.6 (5)	N2—C7—C12	107.9 (6)
N3—N2—Ag1^i^	119.5 (4)	N2—C7—C8	131.5 (6)
C7—N2—Ag1^i^	131.0 (4)	C12—C7—C8	120.6 (6)
N2—N3—N4	107.3 (5)	C9—C8—C7	116.4 (6)
N3—N4—C12	111.1 (5)	C9—C8—H8	121.8
N3—N4—C1	119.3 (5)	C7—C8—H8	121.8
C12—N4—C1	129.4 (5)	C8—C9—C10	122.3 (7)
N4—C1—C2	112.5 (5)	C8—C9—H9	118.9
N4—C1—H11	109.0	C10—C9—H9	118.8
C2—C1—H11	109.1	C11—C10—C9	122.2 (6)
N4—C1—H12	109.2	C11—C10—H10	118.9
C2—C1—H12	109.1	C9—C10—H10	118.9
H11—C1—H12	107.8	C10—C11—C12	115.5 (6)
C3—C2—N1	123.0 (6)	C10—C11—H1	122.3
C3—C2—C1	121.2 (6)	C12—C11—H1	122.2
N1—C2—C1	115.9 (6)	N4—C12—C11	132.9 (6)
C2—C3—C4	120.1 (7)	N4—C12—C7	104.1 (5)
C2—C3—H3	119.9	C11—C12—C7	123.0 (6)
C4—C3—H3	119.9	O3—Cl1—O2	111.3 (7)
C5—C4—C3	117.3 (7)	O3—Cl1—O1	107.7 (5)
C5—C4—H4	121.4	O2—Cl1—O1	108.7 (4)
C3—C4—H4	121.2	O3—Cl1—O4	110.1 (6)
C4—C5—C6	119.7 (7)	O2—Cl1—O4	109.2 (4)
C4—C5—H5	120.1	O1—Cl1—O4	109.7 (5)
			
N2^i^—Ag1—N1—C6	53.0 (7)	C4—C5—C6—N1	1.5 (11)
N2^i^—Ag1—N1—C2	−124.0 (6)	N3—N2—C7—C12	−1.4 (7)
C7—N2—N3—N4	0.4 (7)	Ag1^i^—N2—C7—C12	178.4 (4)
Ag1^i^—N2—N3—N4	−179.4 (4)	N3—N2—C7—C8	−178.6 (7)
N2—N3—N4—C12	0.8 (7)	Ag1^i^—N2—C7—C8	1.1 (11)
N2—N3—N4—C1	176.1 (5)	N2—C7—C8—C9	178.0 (7)
N3—N4—C1—C2	−89.7 (7)	C12—C7—C8—C9	1.1 (10)
C12—N4—C1—C2	84.6 (8)	C7—C8—C9—C10	−1.5 (11)
C6—N1—C2—C3	−1.1 (9)	C8—C9—C10—C11	1.2 (12)
Ag1—N1—C2—C3	175.9 (5)	C9—C10—C11—C12	−0.3 (11)
C6—N1—C2—C1	178.0 (5)	N3—N4—C12—C11	177.9 (7)
Ag1—N1—C2—C1	−4.9 (7)	C1—N4—C12—C11	3.3 (11)
N4—C1—C2—C3	−106.9 (7)	N3—N4—C12—C7	−1.6 (7)
N4—C1—C2—N1	74.0 (7)	C1—N4—C12—C7	−176.2 (6)
N1—C2—C3—C4	1.1 (10)	C10—C11—C12—N4	−179.6 (7)
C1—C2—C3—C4	−178.0 (6)	C10—C11—C12—C7	−0.2 (10)
C2—C3—C4—C5	0.2 (11)	N2—C7—C12—N4	1.8 (7)
C3—C4—C5—C6	−1.4 (11)	C8—C7—C12—N4	179.4 (6)
C2—N1—C6—C5	−0.2 (10)	N2—C7—C12—C11	−177.8 (6)
Ag1—N1—C6—C5	−177.4 (5)	C8—C7—C12—C11	−0.2 (10)

Symmetry codes: (i) −*x*+1, −*y*+1, −*z*+1.

### Hydrogen-bond geometry (Å, °)

**Table:**

*D*—H···*A*	*D*—H	H···*A*	*D*···*A*	*D*—H···*A*
C1—H11···O3^i^	0.97	2.31	3.264 (15)	168
C1—H12···O2^ii^	0.97	2.58	3.415 (11)	144
C3—H3···O1^ii^	0.93	2.60	3.512 (10)	168
C11—H1···O1^ii^	0.93	2.56	3.481 (11)	170

Symmetry codes: (i) −*x*+1, −*y*+1, −*z*+1; (ii) −*x*, *y*+1/2, −*z*+1/2.
